# Correction: Correlation between hospitalized patients’ demographics, symptoms, comorbidities, and COVID-19 pandemic in Bahia, Brazil

**DOI:** 10.1371/journal.pone.0248458

**Published:** 2021-03-11

**Authors:** Márcio C. F. Macedo, Isabelle M. Pinheiro, Caio J. L. Carvalho, Hilda C. J. R. Fraga, Isaac P. C. Araujo, Simone S. Montes, Otávio A. C. Araujo, Lucas A. Alves, Hugo Saba, Márcio L. V. Araújo, Ivonete T. L. Queiroz, Romilson L. Sampaio, Márcia S. P. L. Souza, Ana Claudia F. N. da Silva, Antonio C. S. Souza

The following information is missing from the Funding statement: the Dean’s Office for Research, Graduation and Innovation at the Federal Institute of Bahia–PRPGI/IFBA (https://portal.ifba.edu.br/prpgi), funding program 2019/2020.

## References

[1] Macedo MCF, Pinheiro IM, Carvalho CJL, Fraga HCJR, Araujo IPC, Montes SS, et al. (2020) Correlation between hospitalized patients’ demographics, symptoms, comorbidities, and COVID-19 pandemic in Bahia, Brazil. PLoS ONE 15(12): e0243966. 10.1371/journal.pone.0243966 33318711 PMC7735911

