# Supplementary material for: Correlation between hospitalized patients’ demographics, symptoms, comorbidities, and COVID-19 pandemic in Bahia, Brazil
Source: PLoS One. 2020 Dec 14;15(12):e0243966. doi: 10.1371/journal.pone.0243966 (PMC7735911; doi:10.1371/journal.pone.0243966)
Supplement: Attachment — Submitted filename: Letter of Rebuttal.pdf [file pone.0243966.s002.pdf]

Ms. Ref. No. : PONE-D-20-29331

Title: Correlation between hospitalized patients' demographics, symptoms, comorbidities, and COVID-19 pandemic in Bahia, Brazil

Associate Editor

Editor: *We note that you have indicated that data from this study are available upon request. PLOS only allows data to be available upon request if there are legal or ethical restrictions on sharing data publicly. For information on unacceptable data access restrictions, please see http://journals.plos.org/plosone/s/data-availability#loc-unacceptable-data-access-restrictions..*

Reply: To comply with the data availability requirements of PLOS One, we have decided to upload the anonymized dataset that we have used in this work as a Supporting Information file of this revised submission. After talking with representatives from the Health Secretary of the State of Bahia, we could see that ethical restrictions were no longer applied on this de-identified version of the dataset.

Changes in Manuscript:

- Supporting Information - S1 Table;

Reviewer #1

Reviewer: *The percentage of deaths should be clearly given in the text early on in the results section.*

Reply: We thank the reviewer #1 for his/her suggestion and, in this revised version of the manuscript, we have added the information that 26.8% of the hospitalized patients diagnosed with COVID-19 died from such a disease in Bahia, Brazil.

Changes in Manuscript:

- Results: Lines 113 - 114;

Reviewer: *Is it possible that better records were provided for patients who were more severe and/or died and many of the incomplete or incorrect records were for milder patients? Because it seems to be a very high death rate.*

Reply: Unfortunately, that is not possible. Regardless of the patient's condition, the Health Secretary of the State of Bahia is not receiving more detailed data, such as laboratory clinical parameters. They are only receiving the types of records that we reported in the "Data Collection" section.

Reviewer: *table 1 is useful because it provides the outcomes, but it is unclear why the percentages don't add up. Are there patients who were still being monitored at the time of submission? In other words, are there unresolved cases that are not indicated here?.*

Reply: As we describe in the legend of Fig 1, we have processed the dataset to exclude patients whose final outcome (death or recovery) was unavailable. In this sense, we would like to state that the percentages add up, but with respect to the final outcome of the patients. For instance, if you look at the "Sex" variable in Table 1, you can see that 39.6% of the patients that died from COVID-19 were female patients, while 60.4% (the complement) of the patients that died were male patients. The same observation is held for the "Race" variable in Table 1. Out of the total of hospitalized patients that recovered from COVID-19, 63.2% were black, 13.4% were white, 13.0% were asian, 10.2% had an unknown race, and 0.2% were indian, adding up a total of 100%. On the other hand, the percentages do not add up for symptoms, comorbidities, and the variables inside the "Others" row, because every patient may report more than one symptom, or suffer from zero to more than one comorbidity, and so on. To make this statement clearer for the reviewer and for the readers, we have added a note in Table 1 to reinforce that categorical binary variables are reported using the pattern: count (relative percentage with respect to the total number of deaths or recoveries).

Changes in Manuscript:

- Table 1 - Legend;

Reviewer: *Also in table 1, what are the numbers in parentheses beside the average age? From the table above it suggests that it should be the n, but that is not possible.*

Reply: Those are the standard deviations. To make it clearer for the reviewer and the readers, we added a note in Table 1 to state that discrete variables in this table are reported using the pattern: mean (standard deviation).

Changes in Manuscript:

- Table 1 - Legend;

Reviewer: *The definition of "race" is not standard and seems politically incorrect in English. For example words such as "yellow" have negative connotations and can be interpreted as racist. I think more standard terms need to be given rather than the colors which might be a direct translation but do not sound appropriate in English.*

Reply: We would like to thank the reviewer for his/her great suggestion, and, indeed, we had provided an inaccurate translation of the terms used in Brazil to describe race. Thanks to his/her comment, we studied more how to describe race using more standard terms, and how to map the terms used in Brazil, to the terms appropriate in the English language. In this sense, we looked up at how the U.S. Census approaches this race question (https://www.census.gov/topics/population/race/about.html), and we could see that terms like: *white*, *black*, *indian*, and *asian*, can be used to describe race in a standard way. In this sense, we have changed Table 1 to merge the term "brown" with the term "black", and replace the term "yellow" by the term "asian". Again, we thank the reviewer for his/her recommendation and we hope to avoid making such mistakes in the future.

Changes in Manuscript:

- Table 1 - Race;
- Fig 6;

Reviewer: *Perhaps in the results the kinds of immunosuppression could be given? Is this due to chronic drug use by those patients or does it also include HIV infections?*

Reply: After consulting representatives from the Health Secretary of the State of Bahia, we can state that immunosuppression, in the provided dataset, may refer to a patient with HIV infection or autoimmune disease. We have added such a statement in the legends of Tables 1 and 3.

Changes in Manuscript:

- Table 1 - Legend;
- Table 3 - Legend;

Reviewer: *It would be helpful since the numbers of low and high risk pregnancy are few, to add a category of total pregnancy to the list? Were pregnant women more likely to be hospitalized, potentially explaining their better outcomes?*

Reply: In the provided dataset, pregnancy was classified as low-risk or high-risk. To help us explain why most of the low-risk pregnant women could recover from COVID-19, we followed the suggestion of the reviewer and estimated that 91.4% of the low-risk pregnants diagnosed with COVID-19 were hospitalized. We believe that, indeed, since pregnant women were more likely to be hospitalized, most of them could recover from COVID-19. We added a brief discussion on this topic in the results section.

Changes in Manuscript:

- Results: Lines 135 - 140;

Reviewer: *For Table 2, significant p-values could be highlighted or otherwise indicated to aid readability.*

Reply: Indeed. To accommodate the suggestion of the reviewer, significant p-values are now written in bold in Tables 1, 2, 3, and 4 of the manuscript. We have also changed the legends of those tables in order to reflect this update.

Changes in Manuscript:

- Tables 1, 2, 3, 4 - Legends and p-value columns;

Reviewer: *For Fig 1. Please use commas rather than decimals in the numbers (e.g. 3.896 -> 3,896).*

Reply: We thank the reviewer for pointing out our mistake. We revised not only Fig 1, but the entire manuscript in order to correct such a mistake.

Changes in Manuscript:

- Abstract;
- Introduction - Lines 40, 42;
- Fig 1;
- Results - Lines 107, 110, 117;
- Table 1;

- Conclusion - Line 282;

Reviewer: *For Figs 3-6 (which were out of order in the file, please correct next time) It would be good to put an indication on the figure of the p-value and/or if it was significant. The figure legends need to contain the statistical test used for all figures..*

Reply: We thank the reviewer for his/her suggestions. We updated the figure legends to indicate the statistical test used to measure the p-values, and whether those p-values were statistically significant. Also, we will be more careful to the order of the figures when uploading them in the submission system.

Changes in Manuscript:

- Legends of Figs 3, 4, 5, and 6;

Reviewer: *The discussion should comment on the high mortality rate of the selected group of patients and how this compares to the overall mortality rate in Brazil. Also, some comparison to studies that were done in other regions where similar or different observations were made would greatly improve the discussion.*

Reply: In this revised version of the manuscript, we have added a new paragraph in the Discussion section to accommodate the suggestion of the reviewer and discuss how the results collected in Bahia, Brazil, are compared to the results reported by related work, mainly in terms of mortality rate for hospitalized patients.

Changes in Manuscript:

- Discussion: Lines 245 - 264;

Reviewer #2

Reviewer: *There are a number of concerns regarding how the analysis was presented/conducted. Given that hospital length of stay and definitive outcome are going to be high-correlated for patients hospitalized for COVID-19, it would seem best to treat length of stay, death, and recovery as competing outcomes and to analyze the data accordingly. I would also like to see more efforts to account for the potential confounding effects that different characteristics might have on each other, possibly using a multivariable regression approach to that analysis.*

Reply: We would like to thank the reviewer #2 for his/her suggestion and, to improve the statistical analysis provided in the manuscript, we have added two new tables: Table 2 reports the results obtained with a multivariable logistic regression performed with the statistically significant variables related to the definitive outcome of the hospitalized patients (Table 1), and Table 4 reports the results obtained with a multivariable linear regression performed with the statistically significant variables related to the length of hospital stay (Table 3). On the basis of the results estimated by those multivariable regressions, we performed a major review in the manuscript in order to present and discuss which variables were estimated to be risk factors associated with an increased risk of mortality or length of hospital stay from COVID-19.

Changes in Manuscript:

- Abstract;
- Statistical analysis: Lines 100 - 104;
- Results: Lines 144 - 153, 196 - 207;
- Discussion: Lines 209 - 214;
- Conclusion: Lines 289, 290, and 292;
- Table 2;
- Table 4;
