# Supplementary material for: Correlation between hospitalized patients’ demographics, symptoms, comorbidities, and COVID-19 pandemic in Bahia, Brazil
Source: PLoS One. 2020 Dec 14;15(12):e0243966. doi: 10.1371/journal.pone.0243966 (PMC7735911; doi:10.1371/journal.pone.0243966)
Supplement: Attachment — Submitted filename: Letter of Rebuttal.pdf [file pone.0243966.s003.pdf]

Ms. Ref. No. : PONE-D-20-29331R1

Title: Correlation between hospitalized patients' demographics, symptoms, comorbidities, and COVID-19 pandemic in Bahia, Brazil

Associate Editor

Dear Associate Editor, in this letter of rebuttal we address the comment provided by Reviewer #1, since Reviewer #2 has stated that all of his/her previous comments have been addressed and he/she has provided no further response. Thank you for your assistance!

Reviewer #1

Reviewer: *The authors have addressed my comments and I only had one minor confusion remaining. Which race category are the authors using for native american populations? I wasn't sure if they used "Indian" for this and if so, it might be better to change to "native american" to avoid confusion with India as a country of origin..*

Reply: We thank the reviewer #1 for his/her suggestion and, indeed, we have used the term "Indian" to describe "Native American". In this revised version of the manuscript, we have followed the suggestion of the reviewer and we changed "Indian" to "Native American" to avoid confusion.

Changes in Manuscript:

- Table 1;
- Table 4;
- Figure 6;
- Supporting Information;
